# Contamination Potentials of Household Water Handling and Storage Practices in Kirundo Subcounty, Kisoro District, Uganda

**DOI:** 10.1155/2019/7932193

**Published:** 2019-03-03

**Authors:** Alexander Agensi, Julius Tibyangye, Andrew Tamale, Ezera Agwu, Christine Amongi

**Affiliations:** ^1^Department of Public Health and Community Medicine, Kampala International University-Western Campus, P.O Box 71, Bushenyi, Uganda; ^2^Department of Microbiology and Immunology, Kampala International University-Western Campus, P.O Box 71, Bushenyi, Uganda; ^3^Department of Microbiology and Immunology, St. Augustine International University, P.O Box 88, Kampala, Uganda; ^4^Department of Medical Microbiology and Immunology, Kabale University School of Medicine KABSOM, P.O Box 317, Kabale, Uganda

## Abstract

**Introduction:**

Waterborne diseases constitute a major public health burden in developing and underdeveloped countries. Consumption of contaminated water causes health risk to the public, and the situation is alarming in rural areas. The objective of this study was to assess the contamination potentials of different house water handling and storage practices in the Kirundo subcounty, Kisoro District, Uganda.

**Material and Methods:**

A cross-sectional and descriptive study in which 344 water samples were collected randomly and analysed for bacteriological contamination, total coliforms (TCs) and *Escherichia coli* per 100 ml, using the Most Probable Number (MPN) technique and reported in terms of CFU/100 ml.

**Results:**

The 43.2% samples from unprotected water sources had total coliforms and 34.1% had *Escherichia coli*. In analysed household drinking water, 25% had total coliforms and 8.7% had *Escherichia coli*. Most drinking water sources were found to have coliform counts above the recommended national and international guidelines. There was a statistically significant difference among water sources with respect to total coliforms and *Escherichia coli* (*p* < 0.05).

**Conclusion:**

The overall results indicated that there is a strong linkage between microbiological water quality and water source sanitation; hence, the protected water source was safer than unprotected water sources. For the unprotected water sources, protection strategies as well as monitoring are recommended for this community.

## 1. Introduction

Access to clean and safe water, good sanitation, and hygiene practices are necessary for a healthy population [1]. Whereas the right to safe drinking water is a human right, one-sixth of the world's population cannot access safe drinking water, predisposing communities to the risk of waterborne diseases [2]. Globally, 700 million people lack access to safe drinking water and half of these are in sub-Saharan Africa, where WHO estimates that 1.8 billion people drink water contaminated with *Escherichia coli*, which is an indicator of faecal contamination [3]. In third world countries, 80% of all diseases are directly related to poor-quality drinking water largely attributed to contaminants originating from unsanitary conditions [4].

Availability of healthy drinking water sources is a main concern in many countries around the world [5]. Contamination of drinking water is one of the greatest public health problems worldwide particularly in developing countries [6] and may be severe at the household level [7, 8]. If public health measures for safe drinking water and adequate sanitation are not enforced, water-related diseases such as cholera, dysentery, salmonellosis, and typhoid can erupt, especially in developing countries where millions of lives are claimed every year [4, 9, 10]. Globally, unsafe water coupled with poor sanitation kills at least 1.6 million children under the age of five years, 84% of them living in rural areas [11, 12].

Drinking water quality in the distribution network is paramount [13], and several studies have reported on microbial safety of water sources [1, 3, 4]. Such studies stimulate programmes aimed at safe water supply to communities, thus improving public health and reducing waterborne disease burden. However, efforts by government and NGOs to provide reliable sources of drinking water get jeopardized by poor handling and storage that contaminate the drinking water after it has been drawn from the source [8]. Findings by Parker et al. [14] with similar studies by Amenu et al. and Schriewer et al. [15, 16] show that household water quality is compromised by storage methods after collection, thus increasing the proportion of people drinking unsafe water. In a study by Raju et al. [7], household water showed progressive contamination during storage, and in this study, 73% stored household water samples were contaminated with enteric bacteria.

While rural areas may not have the necessary facilities to process water to be fit for human consumption, abundance of natural pure spring water may be a way to make up for living in water treatment resources poor setting. Protected water may not necessarily be significantly contaminated to pose a public health risk, but the handling from the source to many homes and the pattern of storage before water is finally consumed pose the main challenge for stakeholders including water consumers, water resource managers, and water storage facilities at communities and household levels. While the total coliforms and *Escherichia coli* in water are known to indicate the hygienic condition and health risks associated with water contamination [1], the magnitude of water contamination associated with handling and storage pattern is not clear. As at the time of this study, we are not aware of any report with details of levels of contaminations associated with handling and storage practices in Kirundo, Kisoro District of Uganda. Some interventions on waterborne disease have provided pipeborne water to supplement the central gravity water source used by dwellers in the study area. However, the available report on water crisis and associated health problems show need for interventions to stakeholders.

Assessment of water quality to ensure microbiological safety is a vital public health function to prevent waterborne diseases. The TC and *E. coli* examination provide indication of the hygienic condition of drinking water and are major tools in the assessment of the health risks to waterborne pathogens [1]. Unfortunately, there is insufficient information on the TC and *E. coli* amounts in the common drinking water sources in Kirundo subcounty, Kisoro District, Uganda. It is based on this premise information gap about water contamination despite interventions that this study sought to assess the contamination potentials of household water handling and storage practices in Kirundo subcounty, Kisoro District, Uganda.

## 2. Materials and Methods

### 2.1. Study Design

A cross-sectional and descriptive study was carried out in Kirundo subcounty, Kisoro District, Uganda, from June to October 2017.

### 2.2. Area of the Study

The study was carried out in Kirundo subcounty which is located in northern Bufumbira, Kisoro District, Uganda. The subcounty borders the Bwindi Impenetrable National Park (BINP) in the north, Bukimbiri and Nyundo subcounties in the east, Nyabwishenya in the west, and Busanza subcounty in the south. Kirundo subcounty is composed of two parishes (Rubuguri and Rutaka) with 29 villages. The study area was selected based on the dense population and health records from the servicing health facility (Rubuguri Health Centre IV) which indicated higher cases of waterborne diseases.

### 2.3. Water Sources

A total of 344 water samples were collected from protected water source (Bikingi Gravity Water), unprotected water sources (i.e., springs, ponds, and rivers), and household drinking water for bacteriological analysis of total coliforms and *Escherichia coli* as an indicator of faecal contamination.

### 2.4. Water Sample Analysis for Total Coliforms and *Escherichia coli*

The water samples were analysed for the presence of total coliforms by using the Standard Methods for the Examination of Water and Wastewater adapted from the American Public Health Association 1999 based on the Most Probable Number (MPN) technique [8].

### 2.5. Data Analysis and Management

Data were collected, coded, entered in EpiData version 3.0, and exported to the Statistical Package for the Social Sciences (SPSS) version 16.0. Quantitative data were analysed using STATA and presented in a tabular form using frequencies and percentages for easy interpretation. To establish relationships, bivariate robust Poisson regression analysis and multivariate robust Poisson regression analysis were done. The results were reported in terms of ratios at 95% confidence interval, and a *p* value of 0.05 was considered significant.

### 2.6. Quality Control

To ensure quality, water samples were transported on ice in a cool box and analysed within 2–4 hours. Additionally, samples were analysed in triplicate using standard methods. Equipment was calibrated before use, and a blank sample and a spiked sample were included in the analysis, and a reference laboratory National Water and Sewerage Corporation (NWSC) was used for validation of the findings.

### 2.7. Ethical Considerations

Approval to conduct the study was obtained from Kampala International University-Western Campus, Institutional Research and Ethics Committee (KIU-WC, IREC), and request of permission from District Health Officer, authorities of Rubuguri water supply scheme, and Park Warden, Bwindi Impenetrable National Park.

## 3. Results

### 3.1. Sociodemographic Characteristics of Study Participants

Majority of the participants (33%) were aged between 41 and 51 years whereas 4% (13/300) were above 61 years. In this study, females were about 30% more than males, with the least number of participants having completed tertiary education. Close to 70% of the participants were peasants. Most of the participants inhabited Kashija and Nyabaremura (Table 1).

### 3.2. Factors Associated with Domestic Water Contamination

The main container used for storage of drinking water was an open jerrican. Over 90% of the participants treated their drinking water, and the most popular method for treating water was boiling. The results showed that 204 (or 68%) of the community members did not wash their hands after visiting the latrine. Details of these findings are shown in Table 2.

### 3.3. Total Coliforms Contamination in Drinking Water at Household Level

From the samples collected, 25% (74/300) of drinking water samples from households were contaminated with total coliform. Household drinking water stored in jars and closed bottles showed more contamination with total coliform than drinking water stored in a jerrican. This was significantly different from water stored in a closed jerrican, as shown in Table 3.

### 3.4. *Escherichia coli* Contamination in Drinking Water at Household Level

The results indicated that 8.7% (26/300) of samples collected from household drinking water were found positive for *Escherichia coli*. Household drinking water stored in jar and bottles were more contaminated with *Escherichia coli*. This was significantly different from water stored in a closed jerrican, as shown in Table 4.

### 3.5. Bivariate Robust Poisson Regression Analysis of Total Coliforms in Household Drinking Water

The results showed that factors associated with total coliform contamination in household drinking water were education, occupation, water storage container, methods of water treatment, days of water storage, and washing hands, as shown in Table 5.

### 3.6. Multivariate Robust Poisson Regression Analysis of Total Coliforms in Household Drinking Water

The results showed that practices associated with total coliform contamination in household drinking water in Kirundo subcounty were age, education, occupation, water storage container, and method of water treatment, as shown in Table 6.

### 3.7. Bivariate Robust Poisson Regression Analysis of *Escherichia coli* in Household Drinking Water

Most factors associated with *Escherichia coli* contamination were occupation, water storage container, methods of water treatment, as shown in Table 7.

### 3.8. Multivariate Robust Poisson Regression Analysis of *Escherichia coli* in Household Drinking Water

Household drinking water contamination with *Escherichia coli* was attributed to the following factors: occupation, education, water storage container, and method of water treatment, as shown in Table 8.

### 3.9. Total Coliform Contamination in Water Sources Available for Domestic Use

The results indicated that pond and river water were 100% contaminated with total coliform (*p* value = 0.001), 8.3% spring samples were contaminated, and tap water was not contaminated with total coliform (*p* value = 0.001), as seen in Table 9.

### 3.10. *Escherichia coli* Contamination in Water Sources Available for Domestic Use

The results showed that pond samples were 100% contaminated with *Escherichia coli*, 66.7% of river samples were contaminated with *Escherichia coli*, and spring and tap water samples were not contaminated with *Escherichia coli* (*p* value = 0.01), as seen in Table 10.

## 4. Discussion

The findings of this study established that 25% of the household water samples intended for drinking was contaminated with total coliforms (TCs) and 8.7% contaminated with *E. coli.* The findings were lower probably as result of some community members using protected water source which was found safe, where majority people (88.7%) were reported treating their household drinking water. The findings were close to [9], where 17.1% of household water samples exceeded acceptable levels of total coliforms and 9.5% exceeded acceptable levels of *E. coli* and raw water consumption assumed to be the primary route of exposure to contaminated water.

At the bivariate analysis, findings indicated that the most significant factors or practices associated with total coliform contamination in household drinking water were education, occupation, water storage container, methods of water treatment, and days of water storage. Findings showed that storage practices of household drinking water that was stored in jar and bottles were highly contaminated with TCs, *p*=0.042 and *p*=0.01, respectively. Participants who used SODIS (solar disinfection) bottles were approximately two times as more likely to have their household water contaminated with total coliform as those who used the boiling method (cPR = 2.17, *p* < 0.05). At the multivariate level, SODIS was more than 1.2 times contaminated with total coliform as compared to the boiling method (*p* < 0.05). This is quite different from the study by Fard et al. [5], where all the bottled drinking water brands analysed were found free from coliforms. Presence of TC in drinking water contravenes the Uganda drinking water standard and World Health Organization drinking water guidelines of 0 CFUs/100 ml. From observation, people stored drinking water in open jars which were dirty and in poor housing and sanitation conditions. Studies from [1, 10] show that there is a progressive contamination of water from source to the point of consumption at the households mostly attributed to dirty collection or storage containers. The study findings also indicated that hand washing after toilet use was 63% less likely to be associated with household drinking water contamination by total coliform (*p*=0.001). However, majority 68% of the people lacked the practice of hand washing. Lack of hygienic practices such as hand washing with soap after visiting latrines is a direct route to household water contamination. Contamination of hands, utensils, food, and clothing can also play a role, particularly when domestic sanitation and hygiene are poor, leading to outbreak of sanitation-related diseases such as cholera and typhoid [11]. It is generally difficult to guarantee good water quality beyond the water source, due to the poor hygiene practices, which result in significant deterioration of the quality of water from the time it is collected from the source to the time it is finally consumed [1, 10].

Approximately 43.2% of samples from water sources were contaminated with TC and 34.1% of samples contaminated with *E. coli*. The findings were lower than that in Manonyane community, Maseru District (Lesotho), where TC was detected in 97% and *E. coli* in 71% of the water samples in unprotected water sources [12]. The findings showed that tap water was not contaminated with TC (*p*=0.001) but within Uganda and WHO guidelines of rural drinking water of 0 CFU/100 ml [3]. Probably, this could be related to the fact that the study was done in a rural setting where piped water was completely free from waste water from sewage which would be a source of contamination in most urban settings. The findings were similar to a study that was done by Raeisia et al. [13], where drinking water samples in the distribution network system had zero total coliform and zero faecal coliform. Findings indicated that all samples collected from ponds were 100% grossly contaminated with both TC and *E. coli* (*p*=0.001), values exceeding the WHO guideline value of TC less than 10 CFUs/100 ml and zero *E. coli* per 100 ml water sample. In a study by Bain et al. [3] and Raeisia et al. [13], the quality of traditional water sources and ponds and rivers is always poor throughout the year but worst during the transition from dry to wet seasons.

All samples analysed from rivers had TC, and 66.7% had *E. coli* (*p*=0.01), and the concentration levels of TC and *E. coli* were above the permissible limits, i.e., 0 CFUs/100 ml (conformity) and 1–10 CFUs/100 ml (low risk) according to World Health Organization drinking water quality guidelines. The rivers Kafuga and Kashasha had a high level of TC counts than the river Karenganyambi. The two rivers are located in valleys, and when it rains, the water runoff from the farms and households end up into these rivers. According to studies [4, 14], heavy rainfalls drain animal and human wastes and other wastes into the water bodies, explaining the high coliform counts observed during wet periods. Water quality is typically compromised following rainfall which brings the coliform alongside [3, 15]. It was noted during data collection that open defecation was still practiced. This implied that when it rained, all this was carried to water bodies leading to contamination. From observations by the researcher, the community shared water sources with animals (cattle). The community collects water for domestic use from the rivers Kafuga and Kashasha from which animals (cattle) take water as well as contaminating the rivers with faeces and urine. The sharing of water sources with animals could partly explain the gross contamination of rivers and ponds. In a study [6, 16, 17], *Escherichia coli* and other groups of coliforms may be present where there has been faecal contamination originating from warm-blooded animals, and the major source of *E. coli* is from animals, since it is a normal flora in animal (cattle) intestines. In a study by Olowe et al. [12], mentioned conditions and practices that seem to contribute to increased total coliform and *Escherichia coli* count as no protection of water sources from livestock faeces.

Springs represent a very significant proportion of improved water supplies provided to communities in developing countries. However, findings revealed 8.3% of the samples had TC (*p*=0.001) with no *E. coli* (*p*=0.01). These findings were lower than that in Manonyane community, Maseru District (Lesotho), where 38% of the samples from unprotected springs had *E. coli* compared to 11% samples in protected springs [12]. Also findings were far lower from suburban areas of Kampala city, where total counts in 90% of the spring samples exceeded the WHO guidelines for drinking water [14]. From these findings, the possible spring water contamination was from sewage. Higabiro spring was contaminated and located in the valley such that when it rained, it would be flooded with heavy mud allowing little water to flow. This forced children to use a stick through the pipe to allow more water to flow which could partly explain the presence of coliforms in the spring water. Studies by Thomas et al. [18] showed that quality of such sources is often very variable and frequently shows gross contamination particularly during the wet seasons. Kashija spring lacked adequate protection and yet inadequate spring protection may lead to contamination of springs with pathogenic bacteria [3, 13, 14]. Absence of a fence allows animals near the well, whose excreta could be a source of contamination.

## 5. Conclusions

The overall results indicated that there is a strong linkage between microbiological (TC and *E. coli*) water quality and water source sanitation; hence, the protected water source was safer than unprotected water sources. The household drinking water was contaminated with both TC and *E. coli*, hence an exposure route to waterborne diseases. Factors that were found to be associated with bacterial contamination in the household water were age, education level, occupation, water storage container, and method of household water treatment. It is possible that community education, adoption, and promotion of appropriate water safety plans can reduce the increasing incidences of waterborne disease in the area.

## Figures and Tables

**Table 1:** Sociodemographic characteristics of study participants.

Variable	Frequency	Percent
*Age (years)*		
21–30	56	18.7
31–40	98	32.7
41–50	99	33.0
51–60	34	11.3
≥61	13	4.33

*Sex*		
Male	107	35.7
Female	193	64.3

*Education*		
Primary	154	51.3
Secondary	110	36.7
Tertiary	36	12.0

*Occupation*		
Peasant	204	68.0
Trader	66	22.0
Civil servant	23	7.7
NGO employee	7	2.3

*Village*		
Kashija	87	29.0
Nyabaremura	78	26.0
Rushaga	30	10.0
Higabiro	75	25.0
Kafuga	30	10.0

**Table 2:** Water and sanitation practices at the household level.

Variable	Frequency	Percent
*Water storage container*		
Closed jerrican	54	18.0
Open jerrican	119	39.7
Jar	50	16.7
Closed bottle	77	25.6

*Treatment*		
Yes	266	88.7
No	34	11.3

*Method of water treatment*		
Boiling	248	93.2
Solar disinfection (SODIS)	16	6.0
Water guard	2	0.8

*Days of water storage*		
One day	10	3.3
Two days	150	50
Three days	131	43.7
Four days	9	3

*Washing hands*		
Yes	96	32
No	204	68

**Table 3:** Total coliforms in household drinking water.

Water storage container	Total coliforms, *n* (%)			
	Absent	Present	cOR	*p* value
Closed jerrican	46 (85.2)	8 (14.8)	1.00	—
Open jerrican	98 (82.4)	21 (17.6)	1.23	0.64
Jar	34 (68.0)	16 (32.0)	2.71	**0.042**
Closed bottles	48 (62.3)	29 (37.7)	3.47	**0.01**

**Table 4:** *Escherichia coli* in household drinking water.

Water storage container	*Escherichia coli*, *n* (%)			
	Absent	Present	cOR	*p* value
Closed jerrican	53 (98.1)	1 (1.9)	1.00	—
Open jerrican	115 (96.6)	4 (3.4)	1.84	0.59
Jar	41 (82.0)	9 (18.0)	11.63	**0.02**
Closed bottles	65 (84.4)	12 (15.6)	9.78	**0.03**

**Table 5:** Bivariate robust Poisson regression analysis of total coliforms in household water.

Variable	Total coliform				
	Absent (*n*=266)	Present (*n*=74)	cPR	95% CI	*p* value
*Age (years)*					
21–30	46 (82.1)	10 (17.9)	1.0	—	—
31–40	68 (69.4)	30 (30.6)	1.71	0.91–3.24	0.097
41–50	78 (78.8)	21 (21.2)	1.17	0.60–2.34	0.62
51–60	25 (73.5)	9 (26.5)	1.48	0.67–3.28	0.33
≥61	9 (69.2)	4 (30.8)	1.72	0.64–4.65	0.28

*Education*					
Primary	112 (72.7)	42 (27.3)	1.0	—	—
Secondary	81 (73.6)	29 (23.4)	0.97	0.64–1.45	0.87
Tertiary	33 (91.7)	3 (8.3)	0.31	0.10–0.93	**0.04**

*Occupation*					
Peasant	149 (73.0)	55 (27.0)	1.0	—	—
Trader	50 (75.8)	16 (24.2)	0.90	0.55–1.46	0.67
Civil servant	20 (87.0)	3 (13.0)	0.48	0.16–1.43	0.19
NGO	7 (100.0)	0 (0.0)	0.05	0.02–0.09	**<0.001**

*Water storage container*					
Closed jerrican	46 (85.2)	8 (14.8)	1.0	—	—
Open jerrican	98 (82.4)	21 (17.6)	1.12	0.56–2.52	0.65
Jar	34 (68.0)	16 (32.0)	2.16	1.01–4.61	**0.046**
Closed bottle	48 (62.3)	29 (37.7)	2.54	1.26–5.13	**0.009**

*Method of water treatment*					
Boiling	198 (79.8)	50 (20.2)	1.0	—	—
SODIS	9 (56.3)	7 (43.8)	2.17	1.18–3.99	**0.013**
Water guard	2 (100.0)	0 (0.0)	0.01	0.003–0.05	**<0.001**

*Days of water storage*					
One day	6 (60.0)	4 (40.0)	1.0	—	—
Three days	87 (66.4)	44 (33.6)	0.84	0.38–1.86	0.67
Four days	5 (55.6)	4 (44.4)	1.11	0.39–3.20	0.85

*Wash hands*					
Yes	85 (88.5)	11 (11.5)	1.0	—	—
No	141 (69.1)	63 (30.9)	0.37	0.20–0.67	**0.001**

**Table 6:** Multivariate robust Poisson regression analysis of total coliforms in household water.

Variable	aPR	95% CI	*p* value
*Age (years)*			
21–30	1.0	—	—
31–40	1.49	0.74–2.99	0.26
41–50	1.27	0.59–2.72	0.54
51–60	1.73	0.70–4.23	0.23
≥61	3.50	1.48–8.25	**0.004**

*Education*			
Primary	1.0	—	—
Secondary	1.20	0.74–1.95	0.46
Tertiary	0.13	0.35–0.44	**0.001**

*Occupation*			
Peasant	1.0	—	—
Trader	1.53	0.91–2.59	0.11
Civil servant	4.62	1.01–21.13	**0.048**
NGO	0.064	0.005–0.27	**<0.001**

*Water storage container*			
Closed jerrican	1.0	—	—
Open jerrican	1.77	0.65–4.80	0.26
Jar	3.72	1.55–8.95	**0.003**
Closed bottle	3.25	1.35–7.79	**0.008**

*Method of water treatment*			
Boiling	1.0	—	—
SODIS	2.67	1.36–5.22	**0.004**
Water guard	1.30	2.56–6.66	**<0.001**

*Days of water storage*			
One day	1.0	—	—
Two days	0.36	0.11–1.15	0.09
Three days	0.95	0.31–2.91	0.93
Four days	1.08	0.26–4.32	0.92

**Table 7:** Bivariate robust Poisson regression analysis of *Escherichia coli* in household water.

Variable	*Escherichia coli*				
	Absent (*n*=266)	Present (*n*=74)	cPR	95% CI	*p* value
*Age (years)*					
21–30	51 (91.1)	5 (8.9)	1.0	—	—
31–40	87 (88.8)	11 (11.2)	1.26	0.46–3.44	0.60
41–50	93 (93.9)	6 (6.1)	0.68	0.22–2.13	0.51
51–60	32 (94.1)	2 (5.9)	0.66	0.13–3.22	0.61
≥61	11 (84.6)	2 (15.4)	1.72	0.37–7.94	0.68

*Education*					
Primary	144 (93.5)	10 (6.5)	1.0	—	—
Secondary	97 (88.2)	13 (11.8)	1.82	0.83–4.00	0.14
Tertiary	33 (91.7)	3 (8.3)	1.28	0.37–4.44	0.69

*Occupation*					
Peasant	187 (91.7)	17 (8.3)	1.0	—	—
Trader	60 (90.9)	6 (9.1)	1.09	0.45–2.66	0.85
Civil servant	20 (87.0)	3 (13.0)	1.56	0.49–4.95	0.45
NGO	7 (100.0)	0 (0.0)	0.04	0.01–0.09	**<0.001**

*Water storage container*					
Closed jerrican	53 (98.1)	1 (1.9)	1.0	—	—
Open jerrican	115 (96.6)	4 (3.4)	1.82	0.21–15.92	**0.03**
Jar	41 (82.0)	12 (15.6)	9.72	1.27–74.25	**0.04**
Closed bottle	65 (84.4)	12 (15.6)	8.42	1.12–63.03	**0.04**

*Method of water treatment*					
Boiling	230 (92.7)	18 (7.3)	1.0	—	—
SODIS	14 (87.5)	2 (12.5)	1.72	0.44–6.80	0.44
Water guard	2 (100.0)	0 (0.0)	0.005	0.001–0.02	**<0.001**

*Days of water storage*					
One day	9 (90.0)	1 (10.0)	1.0	—	—
Two days	143 (95.3)	7 (4.7)	0.47	0.06–3.44	0.46
Three days	115 (87.8)	16 (12.2)	1.22	0.19–8.32	0.84
Four days	7 (77.8)	2 (22.2)	2.22	0.24–20.64	0.48

*Washing hands*					
Yes	93 (96.9)	3 (3.1)	1.0	—	—
No	181 (88.7)	23 (11.3)	0.28	0.09–0.90	**0.033**

**Table 8:** Multivariate robust Poisson regression analysis of *Escherichia coli* in household water.

Variable	aPR	95% CI	*p* value
*Education*			
Primary	1.0	—	—
Secondary	2.96	1.10–7.97	**0.03**
Tertiary	0.34	0.86–1.31	0.12

*Occupation*			
Peasant	1.0	—	—
Trader	2.27	0.85–6.06	0.10
Civil servant	20.27	3.38–121.40	**0.001**
NGO	0.025	0.02–0.28	**<0.001**

*Water storage container*			
Closed jerrican	1.0	—	—
Open jerrican	0.72	0.67–861	0.79
Jar	14.44	1.54–135.15	**0.02**
Closed bottle	5.26	0.63–43.62	0.12

*Method of water treatment*			
Boiling	1.0	—	—
SODIS	1.49	0.67–3.32	0.33
Water guard	5.22	4.23–7.41	**<0.001**

**Table 9:** Total coliform contamination in water sources.

Variable	Total coliform, *n* (%)		
Water sources	Absent	Present	*p* value
Spring	11 (91.7)	1 (8.3)	**0.001**
Tap	14 (100.0)	0 (0.0)	**0.001**
Pond	0 (0.0)	9 (100.0)	**0.001**
River	0 (0.0)	9 (100.0)	**0.001**

**Table 10:** *Escherichia coli* contamination in water sources.

Variable	*Escherichia coli*, *n* (%)		
Water sources	Absent	Present	*p* value
Spring	12 (100.0)	0 (0.0)	**0.01**
Tap	14 (100.0)	0 (0.0)	**0.01**
Pond	0 (0.0)	9 (100.0)	**0.01**
River	3 (33.3)	6 (66.7)	**0.04**

## Data Availability

The research data in the tables used to support the findings of this study are included within the article.
